# Data-Independent Acquisition Proteomics Identifies Plasma Prostaglandin-H2 D-Isomerase as an Early Diagnostic Biomarker for STEMI and NSTEMI

**DOI:** 10.1016/j.mcpro.2025.100996

**Published:** 2025-05-23

**Authors:** Hong-Mei Xue, Hai-Tao Hou, Yu Song, Huan-Xin Chen, Yun-Qiang Zhang, Wen-Tao Sun, Jie Zhou, Xiao-Lin Zhou, Na Sun, Qin Yang, Guo-Wei He

**Affiliations:** 1 Department of Cardiovascular Surgery, The Institute of Cardiovascular Diseases, TEDA International Cardiovascular Hospital, Tianjin University & Chinese Academy of Medical Sciences, Tianjin, China; 2 Tianjin Key Laboratory of Molecular Regulation of Cardiovascular Diseases and Translational Medicine, Tianjin, China; 3 The Institute of Cardiovascular Diseases, TEDA International Cardiovascular Hospital, Tianjin University & Chinese Academy of Medical Sciences, Tianjin, China; 4 Department of Physiology, Hebei Medical University & Hebei Key Laboratory of Cardiovascular Homeostasis and Aging, Shijiazhuang, Hebei, China; 5 Cardiac Intensive Care Unit, TEDA International Cardiovascular Hospital, Tianjin, China; 6 Emergency Unit, TEDA International Cardiovascular Hospital, Tianjin, China

**Keywords:** acute coronary syndrome, STEMI, NSTEMI, biomarkers, data-independent acquisition proteomics, prostaglandin-H2 D-isomerase

## Abstract

Myocardial infarction (MI), including ST-segment elevation myocardial infarction (STEMI) and non-ST-segment elevation myocardial infarction (NSTEMI), remains a leading cause of death worldwide. This study aimed to identify the early diagnostic biomarkers for STEMI and NSTEMI. Plasma samples from 386 patients were classified into four groups: control (CON) (n = 62), unstable angina (UA) (n = 62), STEMI (n = 182), and NSTEMI (n = 80). The protein profiles were analyzed using data-independent acquisition (DIA)-based proteomics to identify differentially abundant proteins (DAPs) followed by bioinformatics analysis and ELISA validation. In STEMI, 93 DAPs were detected. Among the selected DAPs that were further validated in a new cohort of patients, prostaglandin-H2 D-isomerase (PTGDS) was elevated at the earliest onset time of STEMI (T1, 1.45 h (95% CI: 1.16–1.73)) or NSTEMI (T1, 1.48 h (95% CI: 0.97–1.98)) while the current biomarkers (hs-TnI, Myo, CK-MB, and BNP) remained within normal ranges. The analysis of diagnostic indices for plasma PTGDS demonstrated a sensitivity of 63.95% and specificity of 65.38% in STEMI, 70% and 71.15% in NSTEMI. Moreover, AUC was 0.61 (95% CI: 0.53–0.69) in STEMI and 0.78 (95% CI: 0.70–0.86) in NSTEMI. The present study demonstrates that in patients with MI, plasma PTGDS increases at an earlier stage of onset time than the current biomarkers, with similar sensitivity and specificity. Therefore, PTGDS has high potential to be developed as an early diagnostic biomarker. In particular, PTGDS might be of greater clinical significance for patients suspected of NSTEMI, for which the biomarker could be more effective in identifying high-risk patients suffering from MI at an early stage.

## Highlights

• Myocardial infarction (MI), including STEMI and NSTEMI, is still the leading cause of death worldwide.
• This study aimed to identify the early diagnostic biomarkers for STEMI and NSTEMI.
• In both patients with STEMI and NSTEMI, plasma PTGDS increases at an earlier stage of onset time than the current biomarkers (hs-TnI, CK-MB, and Myo) with similar sensitivity and specificity.
• PTGDS has high potential to be developed as an early diagnostic biomarker with high sensitivity and specificity in patients with MI.

## Graphical Abstract

**Figure:**
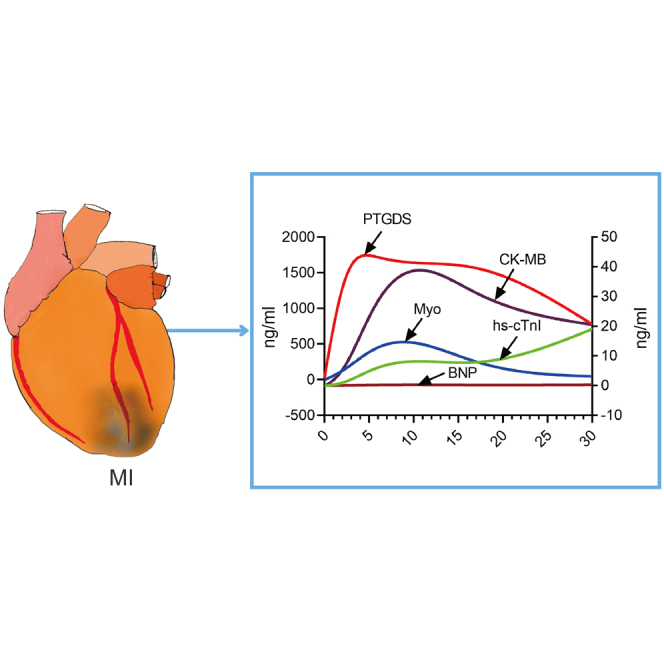

PTGDS increases at an earlier onset time of chest pain than the current biomarkers, indicating that PTGDS is a highly sensitive early biomarker for diagnosis of MI. PTGDS and Myo are plotted on the left Y axis while CK-MB, hs-TnI, and BNP are on the right Y axis.

## In Brief

This proteomics study with new cohort validation identifies prostaglandin-H2 D-isomerase (PTGDS) as a potential early diagnostic biomarker for myocardial infarction (MI), including both ST-elevated MI (STEMI) and non–non-ST-elevated MI (NSTEMI). PTGDS levels rise earlier than the current three biomarkers (hs-TnI, Myo, CK-MB, and BNP) with comparable sensitivity and specificity. In particular, PTGDS has a higher early diagnostic value for NSTEMI. These findings highlight PTGDS as a promising clinical biomarker for early MI detection, especially in patients with NSTEMI.

Cardiovascular disease is the leading cause of death worldwide. Acute coronary syndrome (ACS) refers to a set of conditions that occur due to decreased blood flow in the coronary arteries. ACS is classified into unstable angina (UA), ST-segment elevation myocardial infarction (STEMI), and non-ST-segment elevation myocardial infarction (NSTEMI) (1). Among them, STEMI accounts for approximately 30% of ACS cases, while NSTEMI accounts for almost 70% (2). Therefore, a prompt and precise diagnosis is critical for the treatment of myocardial infarction (MI) (3).

The signs and symptoms were similar in STEMI and NSTEMI. The diagnosis of STEMI and NSTEMI is not differentiated until an electrocardiogram (ECG) has been completed (4). The combination of chest pain, abnormalities in 12-lead ECG, and elevation in cardiac biomarkers such as myoglobin (Myo), creatine kinase MB (CK-MB), and cardiac troponin T (cTnT) or cardiac troponin I (cTnI) contributed to the diagnosis of MI (5). However, the chest pain is often atypical or absent, and the changes in ECG are frequently nonspecific or absent in STEMI. In particular, the initial ECG in NSTEMI might be normal (4). Therefore, cardiac biomarkers have become an important diagnostic tool, even though these biomarkers are elevated in response to cardiac cell injury and may not signal actual infarction (6). The American College of Cardiology (ACC)/American Heart Association (AHA) guidelines recommend troponin as the only cardiac biomarker in patients suspected of MI (7). However, if the initial troponin levels are negative, MI is still suspected (4). Thus, the need for the discovery of new biomarkers for the diagnosis of MI, especially for early diagnosis, remains unmet.

The identification of new biomarkers might depend on the complementary power of genetics, transcriptional profiling, proteomics, and metabolomics (8, 9). Among these, proteomics has emerged as a promising tool and can help identify biomarkers associated with the diagnosis, stage, prognosis, and treatment of diseases, including cardiovascular disease (10, 11, 12). Although plasma proteomic profiling of patients with acute myocardial infarction (AMI) previously identified several biomarker candidates, such as fatty acid-binding proteins, pigment epithelium-derived factors, and several others (1), there remains a clinical need for the identification of new biomarkers with specificity and sensitivity to enhance diagnostic accuracy for MI, particularly NSTEMI. Data-independent acquisition (DIA) ensures the sampling of all peptides within the selected mass ranges, comprehensively and repeatedly analyzing every peptide in a protein digest to produce a complex set of mass spectra (13). DIA has emerged as a powerful approach for large-scale proteomics studies, offering advantages in reproducibility, sensitivity, and quantitative accuracy compared to traditional data-dependent acquisition (DDA) methods (14). As a result, DIA has evolved into a next-generation strategy for high-throughput quantitative proteomics (15).

The present study was designed to identify new biomarkers for the diagnosis of patients with STEMI and NSTEMI, and using DIA proteomic method as well as further validation in a new cohort of patients with the expectation of high sensitivity and specificity.

## Experimental Procedures

### Patients

A total of 386 patients were enrolled from May 2019 to May 2022 at TEDA International Cardiovascular Hospital, Tianjin, China. All human studies were conducted in accordance with the ethical principles outlined in the Declaration of Helsinki. The study was approved by the Institutional Review Board (IRB) of TEDA International Cardiovascular Hospital (Approval No. [2020]-0528-3), and informed consent was obtained from all participants. All patients were fully informed about all aspects of the study. To ensure patient confidentiality, an independent sample library was established. Each blood sample was labeled with a unique QR code containing the patient’s name, medical record number, diagnosis, and other basic information. This system allowed traceability of samples through the hospital information system (HIS system) while maintaining patient privacy and confidentiality.

STEMI is defined as MI in patients with chest discomfort or other ischemic symptoms, accompanied by new ST-segment elevations in two contiguous leads or new bundle branch blocks with ischemic repolarization patterns. Patients presenting without ST-segment elevation but with elevated cardiac biomarkers were designated as having NSTEMI (16). Patients with symptoms suggestive of cardiac ischemia but without ST-segment elevation or elevated biomarker levels can be diagnosed as having UA (5, 16). The inclusion and exclusion criteria for patients with STEMI, NSTEMI, and UA were based on the guidelines outlined by the 2017 European Society of Cardiology (ESC) (17). The control subjects were recruited from patients who underwent conventional coronary angiography for atypical chest discomfort and were found to have normal coronary arteries (18). All patients received treatment and management under the supervision of their attending physicians. The clinical information was collected, including age, gender, body mass index (BMI), risk factors such as hypertension, diabetes, dyslipidemia, and smoking status. The vessel lesion and laboratory data, including relevant biochemical and biomarker measurements, were also collected.

### Blood Sample Collection and Time Distribution

Blood samples were collected from the Emergency Department at the time of the patient’s arrival and from the Cardiac Intensive Care Unit (CICU) on the morning of Day 1 for the STEMI, NSTEMI, UA, and CON (Control) groups. The time points (T1, T2, T3, and T4) were defined based on the duration (in hours) after the onset of chest pain. T1 was defined as 0<T1≤2 h after the onset of the chest pain; 2< T2≤8 h; 8<T3≤24 h; T4>24 h. Patients in four groups (CON, UA, STEMI, and NSTEMI) were age- and gender-matched to ensure comparability. Blood samples in the CON group were collected from patients undergoing normal coronary angiography. Blood samples were centrifuged at 4 °C, 3000 rpm for 15 min, and the plasma was collected and stored at −80 °C for subsequent analysis.

### Experimental Design and Statistical Rationale

The schematic study flowchart is presented in Figure 1. It included a three-phase analytical strategy: Discovery Phase, Validation Phase, and Clinical Evaluation. In the discovery phase, 30 plasma samples were randomly selected from CON, UA, and STEMI groups (10 in each group) at T2 time point for DIA-based biomarker screening. Collected samples were processed through protein extraction, trypsin digestion, and desalting before liquid chromatography-mass spectrometry (LC-MS/MS) analysis. The DIA-mass spectrometry (MS) raw data (SWATH 2.0, ±20 ppm) were matched to a DDA spectral library and quantified.

**Fig. 1:**
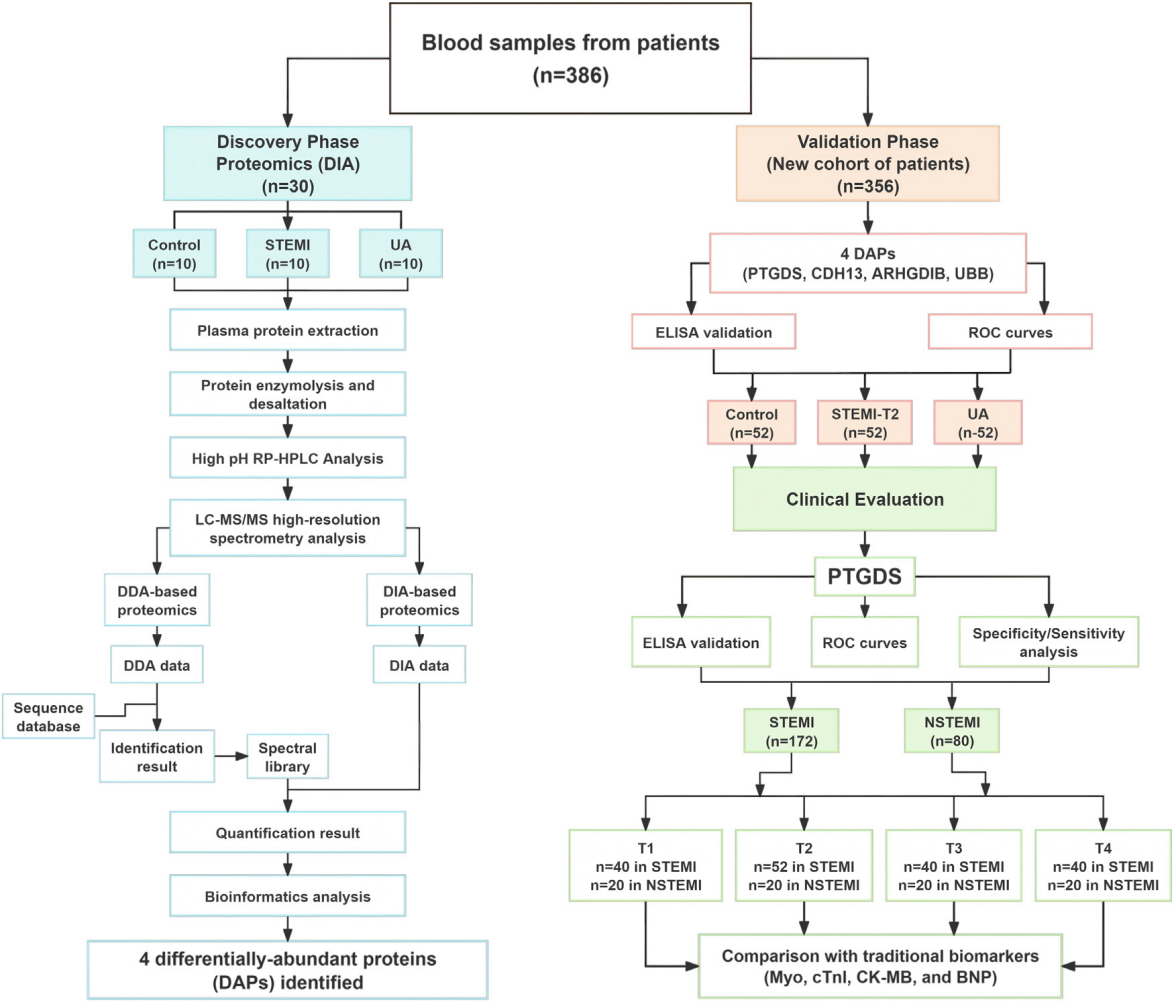
**Schematic flow chart of the data-independent acquisition (DIA) proteomic study**.

In the validation phase, we initially screened 156 samples (52 each from CON, UA, and STEMI groups at T2) for four candidate proteins. PTGDS underwent comprehensive validation across all four timepoints in STEMI (n = 172) and NSTEMI (n = 80), while the other candidates were validated at T2 only.

To evaluate the diagnostic potential of PTGDS, we performed ROC analysis (AUC comparison) and time-dependent sensitivity analysis against conventional biomarkers. These comprehensive statistical approaches demonstrated PTGDS's diagnostic performance for STEMI/NSTEMI cases across all timepoints.

### Preparation of Plasma Sample

DIA-MS was performed by Shanghai Lu-Ming Biotech Co Ltd. The high-abundance proteins were removed using a depletion spin column (85165, Thermo Fisher Scientific) following the manufacturer’s instructions. The resulting protein solution was centrifuged at 12,000*g* for 10 min. The supernatant was collected to quantify the protein concentration using bicinchonininc acid (BCA) assay kit (23227, Thermo Fisher Scientific). The quantified protein samples were aliquoted and stored at −80 °C. The sample proteins were separated on a 12% SDS-PAGE gel. The separation gel was stained with Coomassie blue. Gel images were acquired using an ImageScanner (ES-1000G, EPSON).

### Protein Enzymolysis and Desaltation

Based on the results of protein quantification, 50 μg of protein extract was used for enzymatic digestion. The samples in each group were diluted and normalized to the same concentration and volume using lysis buffer. Dithiothreitol (DTT) was added to the protein solution to achieve a final concentration of 4.5 mmol/l and incubated at 55 °C for 30 min, then cooled on ice to room temperature. The corresponding volume of iodoacetamide was added to reach a final concentration of 9 mmol/l, and the mixture was incubated in the dark at room temperature for 14 min. Then, six volumes of acetone were added to precipitate the proteins and left at −20 °C overnight. After precipitation, the samples were centrifuged at 8000*g* for 10 min at 4 °C. The precipitate was collected, and acetone was volatilized for 2 to 3 min. Then, 100 μl triethylammonium bicarbonate (TEAB) was added to reconstitute the pellet, 1 mg/ml Trypsin-TPCK was added, and the mixture was digested at 37 °C overnight. The reaction was terminated by acidification with phosphoric acid (pH adjusted to 3). Finally, the digested peptides were desalted using a C18-reverse-phase solid-phase extraction (SPE) column (60309-001, Thermo Fisher Scientific) following the manufacturer’s instructions.

### High pH Reverse-Phase High Performance Liquid Chromatography (RP-HPLC) Analysis

Each sample was mixed with iRT standard peptides (Biognosys, Thermo Fisher Scientific) to serve as an internal standard. Reverse-phase (RP) separation was performed by an Agilent 1100 HPLC System (Agilent). An Agilent Zorbax Extend RP column (5 μm, 150 mm × 2.1 mm) was used for peptide separation. Mobile phases A (2% acetonitrile-H_2_O) and B (90% acetonitrile-H_2_O) were adjusted to pH 10 and used for RP gradient. Tryptic peptides were separated at a flow rate of 250 μl/min and monitored at 210 nm and 280 nm. The eluent was collected in a numbered centrifugal tube (1–10) at 1-min intervals from 10 to 50 min. The collection process was repeated in the same order until gradient elution was completed. Finally, the separated peptide fractions were lyophilized and stored for subsequent mass spectrometry (MS) analysis.

### LC-MS/MS Analysis

The lyophilized peptides were analyzed by a Q-Exactive HF mass spectrometer (Thermo Fisher Scientific) equipped with a Nanospray Flex ion source (Thermo Fisher Scientific). Samples were loaded and separated by a C18 column (50 cm × 75 μm) on an EASY-nLC 1200 system (Thermo Fisher Scientific). The flow rate was 300 nl/min and linear gradient was 90 min (0∼60 min, 8–25% B; 60∼79 min, 25–45% B; 79 ∼ 80 min, 45–100% B; 80∼90 min, 100% B; mobile phase A = 0.1% formic acid (FA) in water and B = 0.1% FA in 80% ACN/20% water). At DDA mode, full MS scans were acquired in the mass range of 350 to 1650 m/z with a mass resolution of 120,000, and the AGC target value was set at 3 × 10^6^ with a maximum injection time of 100 ms. The 20 most intense peaks in MS were fragmented with higher-energy collisional dissociation (HCD) with a collision energy of 27. MS/MS spectra were obtained with a resolution of 30,000 with an AGC target of 2 × 10^5^ and a maximum injection time of 80 ms. The Q Exactive HF dynamic exclusion was set for 40.0 s and run under positive mode. At DIA mode, full MS scans were acquired in the mass range of 350 to 1250 m/z with a mass resolution of 120,000, and the AGC target value was set at 3 × 10^6^. The 32 acquisition windows in MS were fragmented with higher-energy collisional dissociation (HCD) with a collision energy of 28, and each acquisition window has 26 m/z. MS/MS spectra were obtained with a resolution of 30,000, with an AGC target of 1 × 10^6^, and a maximum injection time was set to auto and run under positive mode.

### Bioinformatics Analysis

A spectral library was generated experimentally using DDA on the Q Exactive instrument. The DDA raw files were searched against the UniProtKB *Homo sapiens* database (2019_12 release, 20,413 protein entries, uniprot-reviewed-*Homo* *sapiens*(human).fasta) using Proteome Discoverer (v2.3) with the Andromeda search engine. Peptides were identified at a 0.01 false discovery rate (FDR) and used to construct the spectral library. This library was then imported into Spectronaut Pulsar (v13.2.190709) for DIA analysis. Trypsin was specified as the protease and up to 2 missed cleavages were allowed to account for incomplete digestion. Alkylation on Cysteine was set as a fixed modification during the database search. Mass tolerances were set to ±20 ppm for precursor ions and 0.02 Da for fragment ions, based on instrument performance validation and plasma sample characteristics. Chromatogram peak shape was used as a scoring parameter in Spectronaut Pulsar. The dot product (dotp) was calculated to assess the similarity between the experimental chromatogram and the expected elution profile from the spectral library. Peptides with a dotp score ≥0.8 were retained for further analysis. Protein-, peptide- and batch-level FDRs were all set at 0.01. For DIA data, precursor- and protein-level Qvalue were set to 0.01 for high-confidence identification. Quantity MS-level was set at MS2. The significance of the DAPs was determined by fold change (FC>1.2 (upregulated) or <0.8 (downregulated)) and adjusted *p*-value (*p* < 0.05, Benjamini-Hochberg method for multiple testing) was used as the cutoff.

Functional annotation and enrichment analysis were performed for Gene Ontology (GO) annotation in three categories, including biological process (BP), cellular component (CC), and molecular function (MF). A *p*-value cutoff of <0.05 was applied to identify significantly enriched terms. The Kyoto Encyclopedia of Genes and Genomes (KEGG) database was used to classify the identified proteins into biological pathways. The *p*-value cutoff for KEGG enrichment analysis was set to *p* < 0.05 to identify significantly enrichment pathways. Functional analyses of protein-protein interaction (PPI) of selected proteins were performed using the STRING database (http://string.embl.de/). The PPI networks were visualized and analyzed using Cytoscape (www.cytoscape.org/). In the network, nodes represent differentially abundant proteins (DAPs), lines between nodes represent interactions between proteins.

### Validation of DAPs

Prostaglandin-H2 D-isomerase (PTGDS, CSB-EL018969HU), Polyubiquitin-B (UBB, CSB-EL025429HU), Rho GDP-dissociation inhibitor 2 (ARHGDIB, CSB-EL002038HU), and Cadherin-13 (CDH13, CSB-E13817h) were selected for validation by enzyme-linked immunosorbent assay (ELISA) based on their significance in the DIA analysis. Commercially available ELISA kits (CUSABIO) were used, and experiments were performed according to the manufacturer’s instructions.

The sensitivity and specificity of each DAP were evaluated using the receiver operating characteristic (ROC) curve analysis. Diagnostic indices, including area under the curve (AUC), sensitivity, specificity, cutoff point, Youden’s index, likelihood ratio positive (LR+), likelihood ratio negative (LR-), positive predictive value (PPV), negative predictive value (NPV) and accuracy were calculated to assess the diagnostic performance of the selected DAP. Proteins with an AUC > 0.7 were considered to have good diagnostic potential.

### Comparison Between Selected Differential Protein and Current Biomarkers

The selected DAP identified in the present study was compared to the current biomarkers for MI, including myoglobin (Myo), creatine kinase MB (CK-MB), high-sensitivity troponin I (hs-TnI), and brain natriuretic peptide (BNP). The comparison was performed to evaluate the sensitivity of the DAP relative to the current biomarkers at the 4 different timepoints (T1-T4). Plasma was collected from the same patients at the 4 timepoints (T1-T4) to ensure consistency in the comparison. The level of the current biomarkers was measured by the Beckman Coulter Immunoassay System (UniCel Dxl 600) from the Department of Clinical Laboratory of TEDA International Cardiovascular Hospital. The levels of DAPs were evaluated by ELISA kits (CUSABIO).

### Statistical Analysis

Continuous variables are expressed as the median and interquartile range (IQR). Categorical variables are presented as the number and percentage. For ELISA validation analysis, the plasma concentration of DAPs was described by mean ± SEM. The onset time of STEMI and NSTEMI was expressed as the mean with its 95% confidence interval (CI). Continuous variables were compared using one-way ANOVA (normally distributed data) or Kruskal-Wallis test (non-parametric data). Post-hoc analyses were performed with Bonferroni correction for ANOVA or Dunn's test for Kruskal-Wallis results. Categorical variables were compared using the chi-squared test. The significance of ELISA results was determined by one-way ANOVA. Correlations between the biomarkers were analyzed using Pearson correlation analysis. The statistical analyses were performed using GraphPad Prism 8.0 (GraphPad Software). A value of *p* < 0.05 was considered statistically significant.

## Results

### Baseline Characteristics of the Patients

The median door-to-bed time for patients with chest pain during the study period was 55.8 min. Baseline characteristics of the patients included in the proteomic study are summarized in Table 1. There were no significant differences in age, gender, body mass index (BMI), or risk factors such as history of hypertension and dyslipidemia among the CON, UA, STEMI, and NSTEMI groups (*p* > 0.05). However, a higher proportion of patients with STEMI and NSTEMI had hypertension, diabetes, and a history of smoking compared to the CON and UA groups (*p* < 0.05).

**Table 1:** Baseline characteristics for patients

	Control (n = 62)	UA (n = 62)	STEMI (n = 182)	NSTEMI (n = 80)	*p* Value
Age, median (IQR)	62.0 (54.8–67.0)	64.5 (58.8–70.2)	66.0 (57.0–71.0)	66.3 (59.0–71.0)	0.3530
Male, n (%)	26 (41.94%)	34 (54.84%)	102 (56.04%)	57 (71%)	0.0058
BMI, kg/m^2^, median (IQR)	24.25 (20.7–26.8)	26.48 (24.92–28.75)	25.95 (23.41–28.69)	24.75 (22.79–27.43)	0.194
Risk factors, n (%)					
Hypertension, n (%)	29 (47%)	25 (40%)	79 (43%)	60 (75%)	<0.001
Diabetes, n (%)	6 (10%)	17 (27%)	104 (57%)	27 (34%)	<0.001
Dyslipidemia, n (%)	9 (14%)	14 (22%)	51 (28%)	20 (25%)	0.1943
Current smoker, n (%)	6 (10%)	10 (16%)	104 (57%)	38 (48%)	<0.0001
Vessel lesion, n (%)					
Single-vessel disease	0 (0%)	20 (32%)	63 (35%)	15 (19%)	0.0338
Multi-vessel disease	0 (0%)	42 (68%)	119 (65%)	65 (81%)	0.0338
Laboratory data					
PLT (10^9^/L)	248.00 (204.00–265.00)	214.00 (178.00–271.50)	206.00 (169.00–248.00)	227.00 (188.00–278.00)	0.0709
Blood glucose (mmol/L)	5.30 (4.90–5.80)	5.90 (4.90–7.10)	7.10 (5.90–8.90)	6.90 (5.50–8.90)	0.0004
ALT (U/L)	19.00 (13.00–26.00)	19.00 (14.00–25.00)	30.00 (21.00–51.50)	24.00 (17.00–35.00)	<0.0001
AST (U/L)	7.00 (15.00–21.50)	17.00 (13.00–21.50)	101.00 (53.50–183.50)	57.00 (35.00–109.00)	<0.0001
AST/ALT	0.90 (0.70–1.32)	0.90 (0.75–1.10)	3.30 (2.20–4.40)	2.60 (1.80–3.60)	<0.001
GGT (U/L)	23.50 (14.50–40.25)	22.00 (18.00–29.50)	23.00 (16.50–36.50)	24.00 (18.00–35.00)	0.3909
TP (g/L)	67.00 (62.25–70.50)	67.00 (63.00–72.00)	66.00 (63.00–69.00)	66.00 (63.00–70.00)	0.6958
TBIL (μmol/L)	11.25 (8.65–13.82)	10.10 (7.850–12.55)	11.35 (8.325–15.78)	11.50 (8.80–16.00)	0.1058
ALP (U/L)	73.00 (59.00–90.00)	67.00 (48.50–84.00)	63.00 (52.00–75.75)	68.00 (56.00–84.00)	0.0182
UREA (mmol/L)	5.20 (4.73–6.20)	5.60 (5.05–6.88)	6.20 (4.900–7.400)	6.20 (5.20–6.40)	0.0687
Uric acid (μmol/L)	305.00 (264.00–343.00)	327.50 (257.80–408.00)	333.00 (270.80–396.00)	332.00 (274.00–393.00)	0.4223
CREA (μmol/L)	58.00 (48.50–69.50)	62.50 (50.25–71.75)	66.00 (58.75–81.00)	65.00 (56.00–76.00)	0.0364
Total cholesterol (mmol/L)	4.50 (3.80–5.45)	4.40 (3.55–5.15)	4.30 (3.90–4.90)	4.40 (3.50–5.20)	0.7701
TG (mmol/L)	1.34 (0.92–1.89)	1.36 (1.09–2.18)	1.30 (0.84–2.12)	1.38 (0.92–1.98)	0.5802
HDL-C (mmol/L)	1.22 (1.03–1.37)	1.02 (0.92–1.16)	0.94 (0.84–1.13)	1.03 (0.86–1.15)	0.0011
LDL-C (mmol/L)	2.71 (2.13–3.55)	2.94 (2.17–3.42)	2.80 (2.30–3.36)	2.89 (2.08–3.66)	0.8298
Length of stay (days)	2.00 (1.75–3.00)	3.00 (2.00–8.00)	8.00 (7.00–9.50)	7.00 (5.00–8.00)	<0.001

Data are expressed as median (IQR) or numbers (percentage). ALP, alkaline phosphatase; ALT, alanine aminotransferase; AST, aspartate aminotransferase; BMI, body mass index; CREA, creatinine; GGT, glutamyltransferase; HDL-C, high density lipoprotein cholesterol; LDL-C, low density lipoprotein cholesterol; PLT, platelet; TBIL, total bilirubin; TG, triglyceride; TP, total protein.

The levels of current biomarkers—Myo, CK-MB, hs-TnI, and BNP—were elevated in the STEMI and NSTEMI groups compared to the CON and UA groups (*p* < 0.05). Detailed values at different timepoints are provided below. The coronary angiography (CAG) revealed the presence of previous or newly developed vessel lesions in the UA, STEMI, or NSTEMI groups (*p* < 0.05). In contrast, no vessel lesions were detected in the CON group.

Plasma levels of glucose, alanine aminotransferase (ALT), aspartate aminotransferase (AST), AST/ALT ratio, and creatinine (CREA) were elevated in STEMI and NSTEMI compared to the UA and CON groups (*p* < 0.05). Conversely, plasma levels of alkaline phosphatase (ALP) and high-density lipoprotein cholesterol (HDL-C) were lower in the STEMI and NSTEMI groups (*p* < 0.05) (Table 1). No significant differences were observed in platelet counts (PLT), γ-glutamyltransferase (GGT), total protein (TP), total bilirubin (TBIL), plasma alkaline phosphatase (ALP), urea, uric acid, total cholesterol, triglyceride (TG), or low-density lipoprotein cholesterol (LDL-C) among the groups (*p* > 0.05). Moreover, the length of hospital stay was longer for patients with STEMI and NSTEMI compared to the CON and UA groups (*p* < 0.05).

### Identification of the DAPs

Out of the total proteins and peptides that could potentially be identified, 479 proteins and 8235 peptide entries were searched against the DDA library. Thereafter, a total of 479 proteins were quantified in human blood plasma using DIA proteomics (supplemental Table S1). Among them, 93 proteins were identified as DAPs based on the fold change (FC) analysis with an adjusted *p* < 0.05. A total of 70 DAPs were identified, with 33 upregulated and 37 downregulated in the STEMI group compared to the CON group. A total of 43 DAPs, with 18 upregulated and 25 downregulated, were identified in the UA group compared to the CON group. In the comparison between STEMI and UA groups, 31 DAPs with 14 upregulated and 17 downregulated were detected (Fig. 2*A*). Among the 93 DAPs, 51 proteins were found to be differentially abundant across multiple groups, as determined by multiple comparison analysis (Fig. 2*B*).

**Fig. 2:**
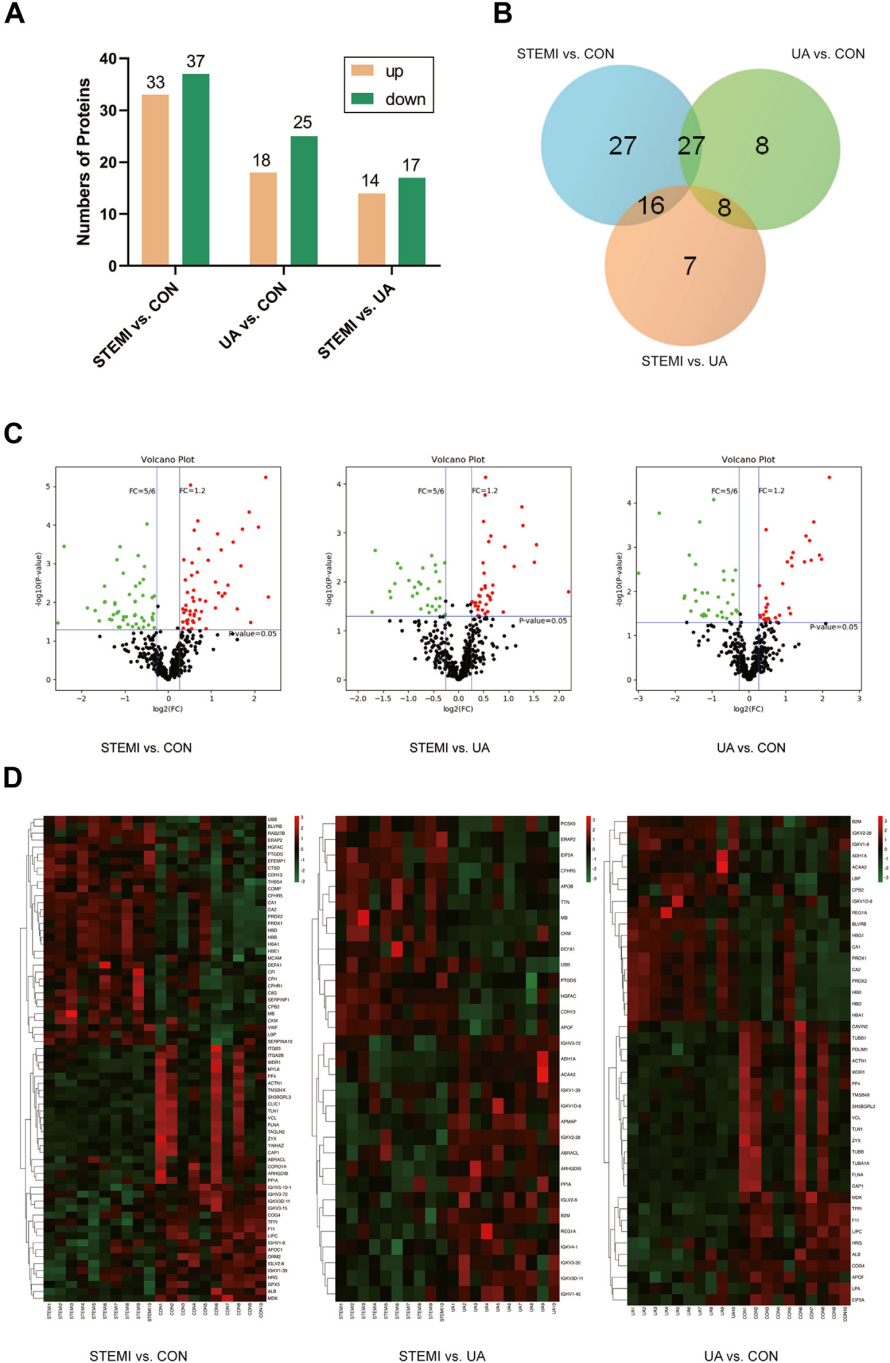

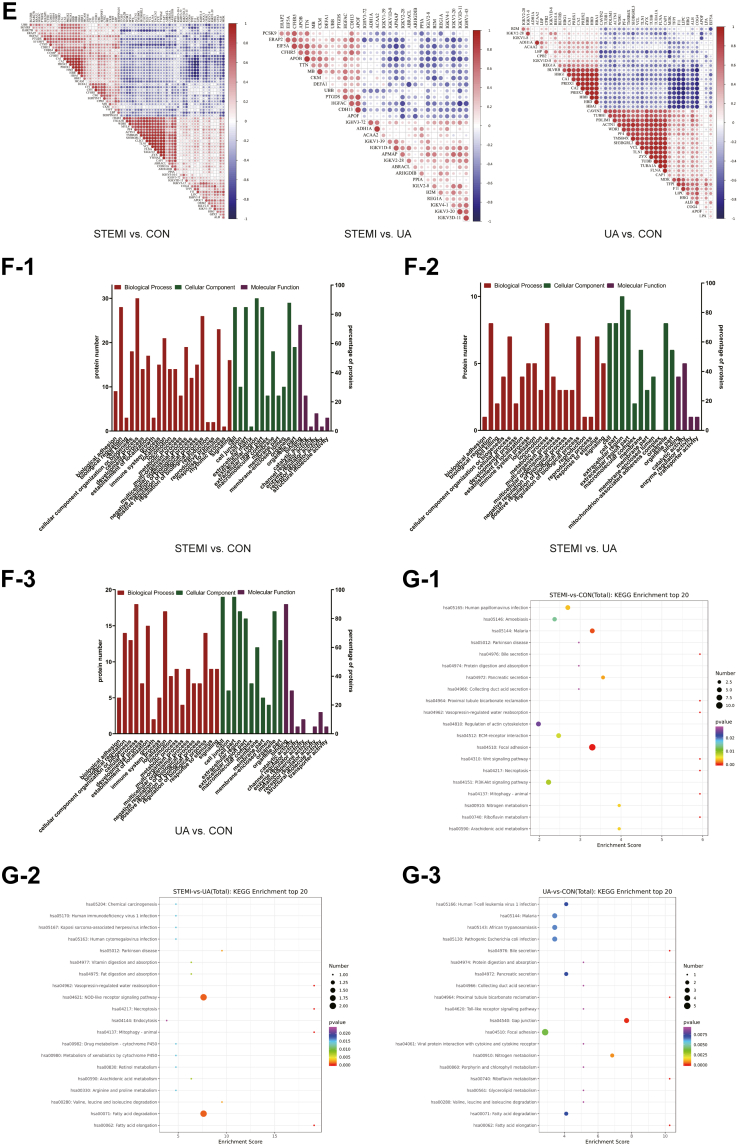

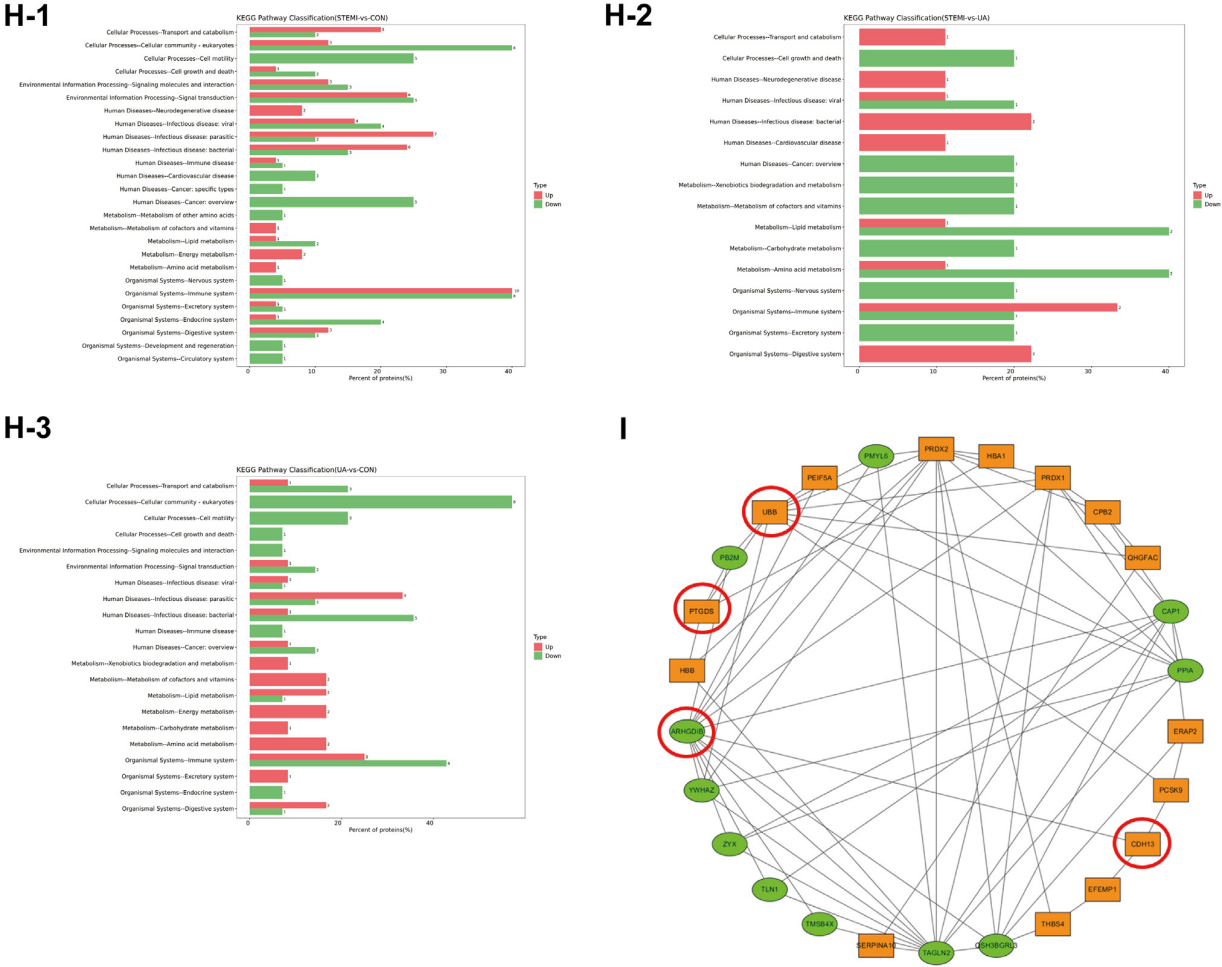
**The identification of the differentially abundant proteins (DAPs), gene ontology (GO) level 2 analysis, and Kyoto Encyclopedia of Genes and Genomes (KEGG) pathway analysis of the DAPs.***A* and *B*, the bar diagram and Venn diagram present the total number of DAPs. *C*, the volcano plot showed the axial distribution of the DAPs. *D*, the heatmap cluster analysis demonstrated the level of the DAPs in STEMI *versus* CON, UA *versus* CON, and STEMI *versus* UA. *E*, Pearson's analysis presents the correlation between the DAPs. *F*-1 to *F*-3, the GO analysis of the DAPs based on their functional classification *G*-1 to *G*-3, the bubble diagram lists the KEGG enrichment top 20 pathways. *H*-1 to *H*-3, KEGG enrichment level 2 analysis of the DAPs. *I*, the interaction of the DAPs was mapped on the protein-protein interaction (PPI) network.

The volcano plots, depicting the total of 93 proteins, including the upregulated or downregulated DAPs, were shown in Figure 2*C*. A heatmap illustrating the expression levels of the DAPs across all groups was presented in Figure 2*D*. The hierarchical clustering analysis demonstrates distinct patterns of protein abundance among the groups. The correlation between the DAPs, as determined by Pearson correlation analysis, was shown in Figure 2*E*.

### Functional Analysis of DAPs

Gene Ontology (GO) level 2 annotation analysis was performed to categorize the DAPs into biological process (BP), cellular component (CC), and molecular function (MF) (Fig. 2*F*). Figure 2*F*-1 shows the comparison of STEMI and CON in 33 DAPs. In BP, the most enriched category was cellular process in 30 proteins (91%). The top category in CC was the extracellular region, involving 30 proteins (91%), while the top category in MF was binding (24 proteins, 73%). The comparison of STEMI *versus* UA is shown in Figure 2*F*-2. Biological regulation and metabolic process ranked first in BP analysis (8 proteins in each, 73%). Extracellular region was the top in CC analysis (10 proteins, 91%), and catalytic activity was the top in MF (5 proteins, 45%). In UA *versus* CON comparison, the top BP, CC, and MF were cellular process (18 proteins, 90%), cell and cell part (19 proteins, 95%), and binding (18 proteins, 90%), respectively (Fig. 2*F*-3). The distribution of the upregulated and downregulated DAPs based on GO level 2 analyses is demonstrated in supplemental Fig. S1*A*.

The top 20 enriched KEGG pathways were displayed in Figure 2*G* as bubble charts. In the STEMI *versus* CON comparison, the most representative pathway included Mitophagy-animal (hsa04137), Proximal tubule bicarbonate reclamation (hsa04964), Bile secretion (hsa04976), Riboflavin metabolism (hsa00740), Vasopressin-regulated water reabsorption (hsa04962), Necroptosis (hsa04217), and Wnt signaling pathway (hsa04310). Each pathway was associated with 1 DAP (Fig. 2*G*-1).

The top pathways in the STEMI *versus* UA comparison included Mitophagy-animal (hsa04137), Vasopressin-regulated water reabsorption (hsa04962), Necroptosis (hsa04217), and Fatty acid elongation (hsa00062). Each group contains 1 DAP (Fig. 2*G*-2). The KEGG enrichment analysis shows the top processes compared between UA and CON were Proximal tubule bicarbonate reclamation (hsa04964), Bile secretion (hsa04976), Riboflavin metabolism (hsa00740), and Fatty acid elongation (hsa00062), encompassing 1 DAP respectively (Fig. 2, Fig. 3*G*-3).

KEGG level 2 process enrichment analysis highlighted the pathways associated with the total proteins and DAPs (supplemental Fig. S1*B*). In the comparison between STEMI and CON, the top pathway for the upregulated proteins (10 proteins) was organismal system-immune system, whereas the top pathways for the downregulated proteins were cellular process-cellular community eukaryotes and organismal system-immune system, each involving 8 proteins (Fig. 2*H*-1). Moreover, the organismal system-immune system was the top pathway for the upregulated proteins (3 proteins) in comparison between STEMI and CON, whereas the top pathway for the downregulated proteins (2 proteins) was lipid metabolism and amino acid metabolism (2 proteins in each metabolism pathway) (Fig. 2*H*-2). The top pathway in UA *versus* CON comparison is human diseases-infectious disease, parasitic, for upregulated proteins (4 proteins) and cellular processes-cellular community eukaryotes for downregulated proteins (8 proteins) (Fig. 2, Fig. 3*H*-3). A protein-protein interaction (PPI) network was constructed for the DAPs identified in the STEMI group (Fig. 2*I*). The network highlights potential functional interactions and regulatory relationships among the DAPs.

### Determination and Validation of the DAPs

The DAPs in plasma among the STEMI, UA, and CON groups were summarized in Table 2. Based on the KEGG enrichment analysis, which identified pathways significantly enriched in DAPs related to inflammation, vascular function, and cellular stress responses that are implicated in STEMI, we selected four DAPs (PTGDS, CDH13, UBB, ARHGDIB) as the candidate diagnostic biomarkers for STEMI. These DAPs were prioritized due to their significant differential abundance across groups, involvement in key STEMI-related pathways, and well-documented roles in cardiovascular diseases (Table 2). The four DAPs showed distinct abundance patterns in both volcano (significance *versus* fold-change) and waterfall (relative ranking) plots (supplemental Fig. S2). ROC curves were generated based on the DIA results to evaluate the diagnostic performance of the candidate biomarkers (Fig. 3*A*). All the values of the area under the curve (AUC) were more than 0.7, indicating significant discriminatory power for distinguishing STEMI from UA and CON. The four candidate biomarkers were further validated using ELISA in a larger cohort of 156 plasma samples (52 samples in each group). The scatter plots showed that the plasma levels of PTGDS (1697.49 ± 121.78 ng/ml in STEMI *versus* 1005.83 ± 88.40 ng/ml in UA, 926.50 ± 80.20 ng/ml in CON, *p* < 0.001) and CDH13 (2.12 ± 0.13 ng/ml in STEMI *versus* 1.68 ± 0.14 ng/ml in UA, *p* < 0.05, *versus* 1.2 ± 0.08 ng/ml in CON, *p* < 0.001) were increased, whereas ARHGDIB (54.07 ± 1.37 pg/ml in STEMI *versus* 64.13 ± 2.04 pg/ml in UA, *p* < 0.001, *versus* 65.23 ± 3.40 pg/ml in CON, *p* < 0.01) was decreased in STEMI *versus* CON or UA, which was consistent with the DIA results. In contrast, the plasma level of UBB in STEMI was higher than that in UA (14.11 ± 0.65 ng/ml in STEMI *versus* 11.52 ± 0.37 ng/ml in UA, *p* < 0.001) but had no significant difference compared to CON (14.11 ± 0.65 ng/ml in STEMI *versus* 13.45 ± 0.67 ng/ml in CON, *p* > 0.05) (Fig. 3*B*).

**Table 2:** Differentially abundant proteins (DAPs) identified in STEMI *versus* CON and UA

Accession	Protein Descriptions	Genes	STEMI *versus* CON		STEMI *versus* UA	
			Log 2 (Fold Change)	*p*-value	Log 2 (Fold Change)	*p*-value
P41222	Prostaglandin-H2 D-isomerase	*PTGDS*	0.67	0.001625	0.67	0.016585
P55290	Cadherin-13	*CDH13*	1.13	0.000165	1.26	0.000289
P0CG47	Polyubiquitin-B	*UBB*	0.92	0.000818	0.62	0.020177
P52566	RhoGDP-dissociation inhibitor 2	*ARHGDIB*	−0.78	0.02216	−0.61	0.013831
A0A0A0MRZ8	Immunoglobulin kappa variable 3D-11	*IGKV3D*-*11*	−0.66	0.003151	−0.72	0.005238
A0A0B4J1Y9	Immunoglobulin heavy variable 3–72	*IGHV3*-*72*	−0.97	0.02355	−1.1616	0.005175
P01709	Immunoglobulin lambda variable 2–8	*IGLV2*-*8*	−1.12	0.000361	−0.92	0.019541
P01597	Immunoglobulin kappa variable 1–39	*IGKV1*-*39*	−1.60	0.029766	−1.73	0.041232
P02144	Myoglobin	*MB*	1.30	0.005835	1.55	0.001729
P06732	Creatine kinase M-type	*CKM*	1.91	0.033447	2.19	0.015968
P59665	Neutrophil defensin 1	*DEFA1*	1.24	0.00675	0.88	0.041304
P62937	Peptidyl-prolyl cis-trans isomerase A	*PPIA*	−1.15	0.017227	−0.75	0.01107
Q04756	Hepatocyte growth factor activator	*HGFAC*	0.71	0.000408	0.60	0.001497
Q6P179	Endoplasmic reticulum aminopeptidase 2	*ERAP2*	0.88	0.048045	1.29	0.000711
Q9BXR6	Complement factor H-related protein 5	*CFHR5*	1.38	0.003604	1.10	0.004836
Q9P1F3	Costars family protein ABRACL	*ABRACL*	−0.94	0.037439	−0.76	0.035908

**Fig. 3:**
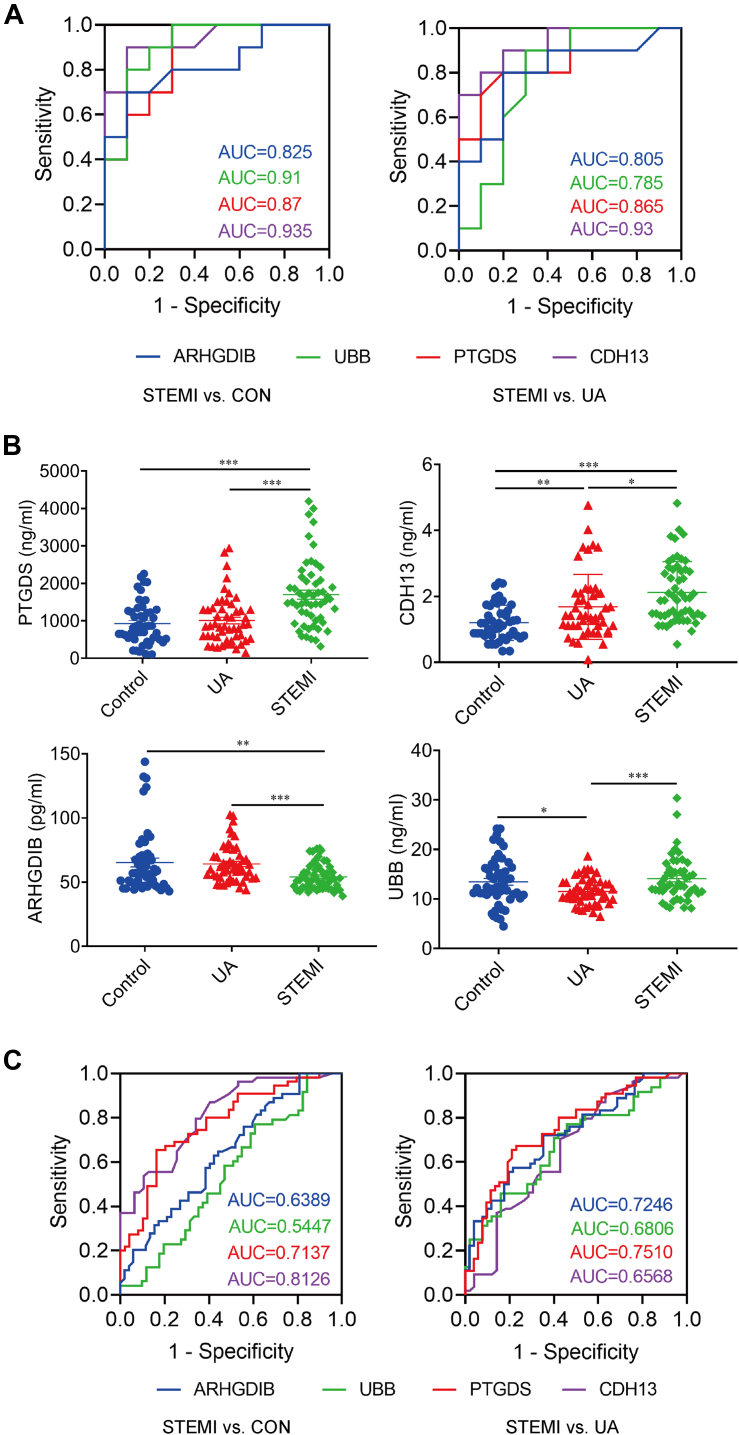
**Receiver–operating characteristic (ROC) curves and ELISA validation of the differentially abundant proteins (DAPs).***A*, the ROC curves of the potential diagnostic biomarkers for STEMI compared with CON and UA based on the DIA results. n = 10 in each group. *B*, the proteins were validated using ELISA. n = 52 in each group. *C*, the ROC curves of the biomarkers were based on the ELISA validation results. n = 52 in each group. ∗*p* < 0.05, ∗∗*p* < 0.01, ∗∗∗*p* < 0.001.

To further evaluate the diagnostic potential of the candidate biomarkers (PTGDS, CDH13, UBB, and ARHGDIB), ROC curves were generated using the ELISA validation results. The value of AUC was 0.77 (95% CI: 0.68–0.86), 0.81 (95% CI: 0.73–0.89), 0.55 (95% CI: 0.43–0.66), and 0.64 (95% CI: 0.53–0.74), respectively, in STEMI *versus* CON (*p* < 0.001). The AUC was 0.75 (95% CI: 0.66–0.84), 0.66 (95% CI: 0.55–0.76), 0.68 (95% CI: 0.58–0.79), and 0.72 (95% CI: 0.63–0.82), respectively, in STEMI *versus* UA (*p* < 0.001). The value of AUC for PTGDS was above 0.7 in STEMI, suggesting its significance as a potential diagnostic biomarker (Fig. 3*C*).

### Sensitivity and Specificity of PTGDS in Patients With STEMI and NSTEMI

The diagnostic performance of PTGDS was evaluated and compared to current biomarkers (Myo, hs-TnI, and CK-MB) in patients with STEMI and NSTEMI using ROC curve analysis (Fig. 4*A*). The optimal cutoff point for PTGDS was 872.70 ng/ml in STEMI and 1087.00 ng/ml in NSTEMI. The sensitivity, specificity, positive predictive value (PPV), negative predictive value (NPV), and accuracy of PTGDS in diagnosis were higher in NSTEMI compared to STEMI (Table 3). In comparison with the current biomarkers, PTGDS demonstrated similar diagnostic indices, including Youden’s index, sensitivity, specificity, likelihood ratio (LR), and AUC, particularly in NSTEMI (Table 3).

**Fig. 4:**
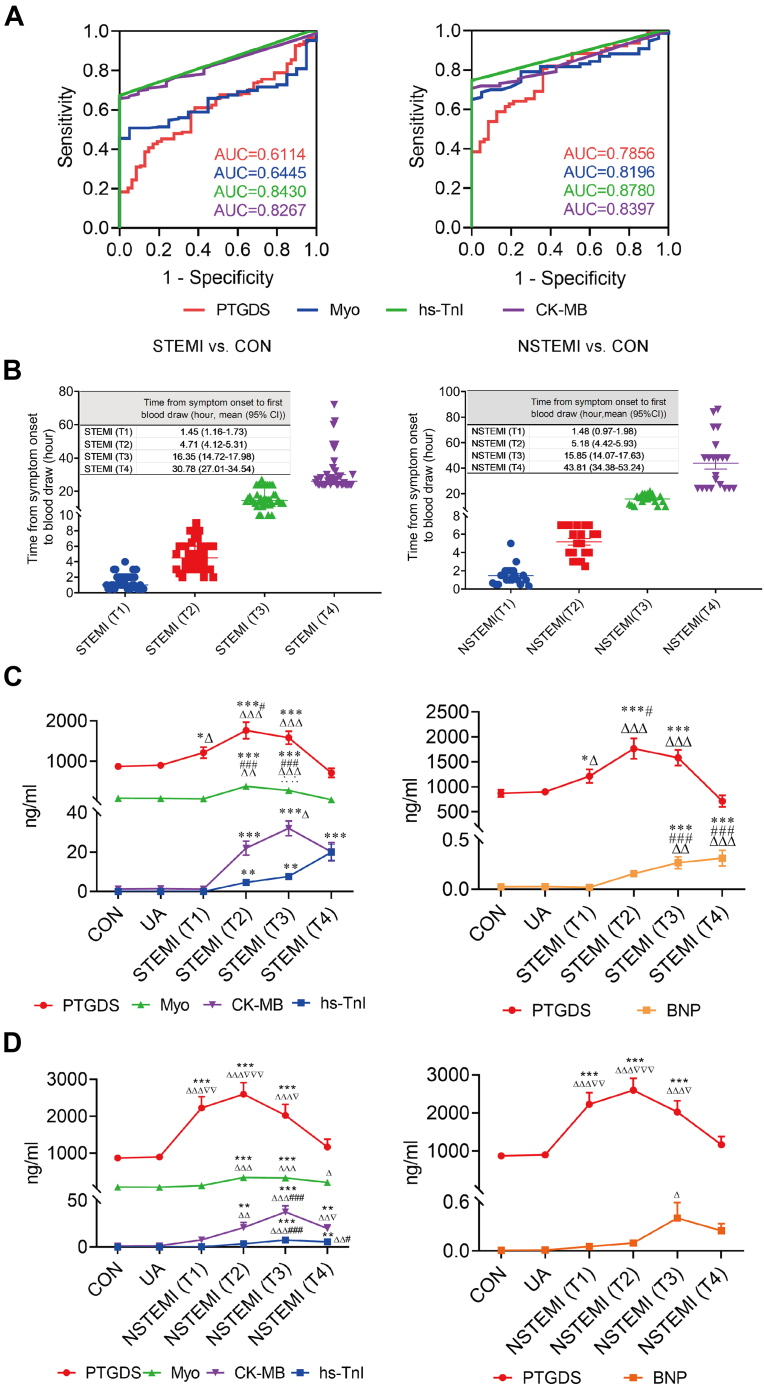
**Analysis of the sensitivity and specificity of PTGDS in STEMI and NSTEMI**. *A*, the ROC curves of PTGDS and current biomarkers. *B*, the timepoint from symptom onset to the first blood draw. *C* and *D*, the concentration of PTGDS and current biomarkers in CON, UA, and STEMI or NSTEMI at four timepoints. ∗*p* < 0.05, ∗∗*p* < 0.01, ∗∗∗*p* < 0.001 *versus* CON, ^Δ^*p* < 0.05, ^ΔΔ^*p* < 0.01, ^ΔΔΔ^*p* < 0.001 *versus* UA, ^#^*p* < 0.05, ^###^*p* < 0.001 *versus* T1, ^∴∴∴^*p* < 0.001 *versus* T2, ^▿^*p* < 0.05, ^▿▿^*p* < 0.01, ^▿▿▿^*p* < 0.001 *versus* T4.

**Table 3:** The diagnostic indices and AUC of PTGDS, Myo, CK-MB and hs-TnI for NSTEMI and STEMI

Group	Variable	Cutoff point (ng/ml)	Youden’s index	Sensitivity	Specificity	LR+	LR-	PPV	NPV	Accuracy	AUC (95% CI)	Earliest changes appear
STEMI	PTGDS	872.70	0.29	63.95%	65.38%	1.85	0.53	85.94%	35.42%	64.29%	0.6114 (0.53–0.69)	T1
	Myo	85.30	0.13	59.30%	53.85%	1.28	0.86	80.95%	28.57%	58.04%	0.64 (0.56–0.72)	T2
	CK-MB	1.55	0.42	74.42%	67.31%	2.28	0.49	88.28%	44.30%	72.77%	0.83 (0.77–0.89)	T2
	hs-TnI	0.05	0.58	67.44%	90.38%	7.01	0.11	95.87%	45.63%	72.77%	0.84 (0.79–0.90)	T2
NSTEMI	PTGDS	1087.00	0.41	70.00%	71.15%	2.43	0.41	78.87%	60.66%	70.45%	0.78 (0.70–0.86)	T1
	Myo	107.50	0.49	76.25%	73.08%	2.83	0.37	81.33%	66.67%	75.00%	0.82 (0.74–0.90)	T2
	CK-MB	1.45	0.42	75.00%	67.31%	2.29	0.49	77.92%	63.64%	71.97%	0.84 (0.76–0.917)	T2
	hs-TnI	0.06	0.66	73.75%	92.31%	9.59	0.08	93.65%	69.57%	81.06%	0.88 (0.82–0.94)	T3

LR+: likelihood ratio positive, LR-: likelihood ratio negative; PPV: positive predictive value; NPV: negative predictive value; AUC, area under the curve; CI: confidence interval.

To evaluate the dynamic changes in PTGDS plasma concentrations over time, we analyzed samples collected at four timepoints following the onset of chest pain in patients with STEMI and NSTEMI. The results are shown in Figure 4*B*. The timepoints from chest pain onset to the first blood draw were 1.45 h (95% CI: 1.16–1.73), 4.71 h (95% CI: 4.12–5.31), 16.35 h (95% CI: 14.72–17.98), and 30.78 h (95% CI: 27.01–34.54) in STEMI (T1), STEMI (T2), STEMI (T3) and STEMI (T4) groups, respectively. As for NSTEMI, the four timepoints were 1.48 h (95% CI: 0.97–1.98), 5.18 h (95% CI: 4.42–5.93), 15.85 h (95% CI: 14.07–17.63), and 43.81 h (95% CI: 34.38–53.24).

All the plasma concentrations of the 4 current biomarkers were obtained via peripheral venipuncture on hospital admission. Table 4 and Figure 4, *C* and *D* demonstrate the plasma levels of PTGDS as well as the 4 current biomarkers hs-TnI, Myo, CK-MB, and BNP in CON, UA, STEMI, or NSTEMI at the 4 timepoints (T1-T4). Importantly, only PTGDS increased at T1 (Fig. 4*C*) in patients with STEMI (1214.01 ± 137.32 ng/ml in STEMI *versus* 871.71 ± 73.50 ng/ml in CON, *p* < 0.05; *versus* 899.53 ± 68.42 ng/ml in UA, *p* < 0.05) and NSTEMI (2228.24 ± 302.35 ng/ml in NSTEMI, *p* < 0.001 *versus* CON or UA), suggesting that among these 5 proteins, PTGDS had the earliest changes as a biomarker to detect STEMI or NSTEMI. The increase of the plasma concentration of PTGDS remains at T2 (1766.22 ± 205.43 ng/ml in STEMI (T2). As to NSTEMI, at T2, PTGDS also remained increased (2598.19 ± 313.09 ng/ml). PTGDS gradually declined thereafter at T3 (1584.91 ± 157.48 ng/ml in STEMI, 2025.64 ± 293.62 ng/ml in NSTEMI) and returned to normal at T4 (713.85 ± 115.06 ng/ml in STEMI, 1165.34 ± 215.14 ng/ml in NSTEMI, *p* > 0.05 *versus* CON or UA). In contrast, the other 4 current biomarkers only increased at T2 or T3.

**Table 4:** Comparison of the plasma PTGDS and current biomarkers among groups

Group		PTGDS (ng/ml)	Myo (ng/ml)	CK-MB (ng/ml)	hs-TnI (ng/ml)	BNP (ng/ml)
Control		871.71 ± 73.50	80.08 ± 6.88	1.33 ± 0.11	0.05 ± 0.002	0.008 ± 0.003
UA		899.53 ± 68.42	77.80 ± 6.12	1.48 ± 0.19	0.06 ± 0.008	0.01 ± 0.003
STEMI	T1	1214.01 ± 137.32^∗Δ^	65.26 ± 3.99	1.28 ± 0.10	0.06 ± 0.008	0.01 ± 0.002
	T2	1766.22 ± 205.43^∗∗∗ΔΔΔ#^	376.90 ± 20.71^∗∗∗ΔΔ###^	22.01 ± 3.56^∗∗∗^	4.65 ± 1.18^∗∗^	0.16 ± 0.03
	T3	1584.91 ± 157.48^∗∗∗ΔΔΔ^	274.00 ± 25.75^∗∗∗ΔΔΔ###∴∴^	32.11 ± 3.78^∗∗∗Δ^	7.65 ± 1.69^∗∗^	0.19 ± 0.05^∗∗∗ΔΔ###^
	T4	713.85 ± 115.06	44.61 ± 8.06	20.16 ± 4.60^∗∗∗^	19.96 ± 3.98^∗∗∗^	0.27 ± 0.07^∗∗∗ΔΔΔ###^
NSTEMI	T1	2228.24 ± 302.35^∗∗∗ΔΔΔ^	124.16 ± 27.32	7.76 ± 2.87	0.35 ± 0.16	0.05 ± 0.01
	T2	2598.19 ± 313.09^∗∗∗ΔΔΔ^	343.69 ± 37.10^∗∗∗ΔΔΔ^	20.97 ± 5.35^∗∗ΔΔ###^	3.47 ± 1.48	0.10 ± 0.02
	T3	2025.64 ± 293.62^∗∗∗ΔΔΔ^	329.60 ± 39.92^∗∗∗ΔΔΔ^	37.82 ± 6.60^∗∗∗ΔΔΔ^	7.67 ± 2.00^∗∗∗ΔΔΔ###^	0.40 ± 0.19^Δ^
	T4	1165.34 ± 215.14	211.20 ± 34.01^Δ^	20.07 ± 3.80^∗∗ΔΔ^	5.65 ± 1.48^∗∗ΔΔ#^	0.24 ± 0.09

BNP, B-type natriuretic peptide; CK-MB, creatine kinase-myocardial band; hs-TnI, high-sensitivity cardiac troponin I; Myo, myoglobin; ^∗^*p* < 0.05, ^∗∗^*p* < 0.01, ^∗∗∗^*p* < 0.001 *versus* Control, ^Δ^*p* < 0.05, ^ΔΔ^*p* < 0.01, ^ΔΔΔ^*p* < 0.001 *versus* UA, ^#^*p* < 0.05, ^###^*p* < 0.001 *versus* T1, ^∴∴∴^*p* < 0.001 *versus* T2.

## Discussion

In this study, we employed a DIA proteomic approach followed by validation in an independent cohort to identify and characterize DAPs in patients with STEMI and NSTEMI. The key findings are as follows: (1) A total of 16 DAPs were detected in patients with STEMI compared to both CON and UA patients; (2) Among the 4 DAPs identified by proteomics (3 upregulated: CDH13, UBB, and PTGDS; 1 downregulated: ARHGDIB), CDH13, PTGDS and ARHGDIB were successfully validated using ELISA; (3) plasma PTGDS increased at an earlier onset time in both STEMI and NSTEMI patients compared to the currently used biomarkers (Myo, CK-MB, hs-TnI, and BNP), with high sensitivity and specificity; (4) The diagnostic performance of PTGDS (sensitivity and specificity) was comparable to that of the current biomarkers, particularly in patients with NSTEMI; and (5) The early rise in PTGDS levels, preceding changes in current biomarkers, highlights its potential value in the early diagnosis of MI, especially in patients with NSTEMI who often lack significant ECG changes.

Both the ACC/AHA guidelines (7) and the European Society of Cardiology (ESC) guidelines (19) emphasize the growing importance of biomarkers in the diagnosis and management of MI. Currently, the primary biomarkers used for the diagnosis of STEMI and NSTEMI are hs-TnI, Myo, and CK-MB (20). cTn is the preferred biomarker for MI diagnosis due to its high myocardial tissue specificity and high clinical sensitivity (21). Detection of a rise and/or fall in cTn levels is essential for diagnosing acute MI (21). CK-MB is the best alternative when the cTn assay is unavailable. As with troponin, an increased CK-MB value is defined as a measurement above the 99th percentile upper reference limit (URL), which serves as the decision level for the diagnosis of MI (22). However, CK-MB is not entirely specific to the heart, as it is also found in amounts up to 1 to 2% of total CK in skeletal muscle (23). Moreover, cTn and CK-MB are typically detectable for myocardial injury 6 h after chest pain onset, and repeated measurements are required at 8-12 h after admission if initial results are negative (24). Myo is the earliest marker, appearing within the first 3h of chest pain onset. However, it is more specific for skeletal muscle injury than for myocardial injury, limiting its diagnostic utility (25). In patients with NSTEMI, ECG changes are often nonspecific or absent, making biomarker-based diagnosis particularly critical (7, 19). The development of novel biomarkers with early diagnostic capabilities could significantly improve the identification of high-risk patients, enabling timely intervention and improving clinical outcomes.

*PTGDS* (OMIM: 176803), also known as lipocalin-type prostaglandin D synthase (L-PGDS) and prostaglandin-D2 synthase (PGDS), is a gene spanning 3600 bp and contains 7 exons (26). PTGDS was initially purified from the rat brain as a 26-kDa glycoprotein (27) and identified as the enzyme responsible for prostaglandin-D2 (PGD2) biosynthesis in the brain. It is also highly expressed in the myocardium, including in cardiomyocytes (28, 29). PTGDS has been extensively identified in autopsy specimens of atherosclerotic plaques of human coronary arteries, with higher expression levels compared to the internal mammary artery or carotid artery (30). Serum PTGDS levels are positively associated with the severity of coronary artery disease, suggesting its potential as a biomarker for atherosclerotic coronary artery disease (30). Further, previous studies showed that PTGDS was found in vascular endothelial cells and secreted into the bloodstream, indicating that endothelial cells might be a major source of PTGDS in serum (31). Serum PTGDS was noted in the coronary circulation of patients with effort angina, and the level was altered after coronary angioplasty (32, 33). In the present study, using the DIA proteomic study, we found that PTGDS levels in the plasma were increased in patients with STEMI compared to patients with UA and CON (FC = 1.59, *p* < 0.05). Bioinformatics analysis revealed that PTGDS is involved in the arachidonic acid metabolism pathway (hsa00590), which is one of the top 20 KEGG level 2 enrichment analyses in comparison of STEMI with UA or CON. A schematic diagram illustrating the role of increased PTGDS in MI, particularly its involvement in the metabolic cascade of arachidonic acid, is demonstrated in supplemental Fig. S3. The increased PTGDS in MI possibly affects the catalysis of PGH2 to PGD2, which subsequently inhibits platelet aggregation (34, 35). This suggests that the elevation of PTGDS in patients with STEMI and NSTEMI may represent a self-protective mechanism, potentially mitigating the progression of myocardial injury.

The present study is the first to identify PTGDS as a sensitive and early biomarker for the diagnosis of STEMI and NSTEMI (Figs. 3 and 4). PTGDS levels increased significantly earlier than the four currently used biomarkers (Myo, CK-MB, hs-TnI, and BNP). The increase in PTGDS was already significant at T1 (1.45 h (95% CI: 1.16–1.73) in STEMI, 1.48 h (95% CI: 0.97–1.98) in NSTEMI). As shown in Figure 4, *C* and *D*, PTGDS was the only biomarker among the five studied to show a significant increase at the early stage (T1) of STEMI or NSTEMI. PTGDS reached its plateau at T2, similar to Myo, but different from the other three biomarkers (CK-MB, hs-TnI, and BNP) that reached the plateau at either T3 (CK-MB) or T4 (hs-TnI and BNP). This early rise and plateau suggest that PTGDS could provide critical diagnostic information during the initial hours of MI, when current biomarkers are not yet elevated. Previous studies suggest that PTGDS is more specific in atherosclerotic plaque of the coronary artery than to other arteries, such as the internal mammary artery and carotid artery (30). In our study, PTGDS did not elevate in patients with UA (Figs. 3 and 4), further supporting its specificity for acute MI. Interestingly, in the comparison of PTGDS to other three current biomarkers, it is rather complex. First, the absolute value of PTGDS was higher than all three other current biomarkers (Fig. 4, *C* and *D* and Table 3), indicating its usefulness as a new biomarker for MI. Second, the sensitivity and specificity of PTGDS were higher than Myo, but slightly lower than CK-MB and hs-TnI in STEMI. Furthermore, the sensitivity of PTGDS was similar to the other three current biomarkers, although the specificity was better than that of CK-MB, similar to Myo, and lower than hs-Tnl. These results suggest that PTGDS is unlikely to replace current biomarkers in clinical practice. Rather, it may serve as a complementary marker, providing additional insight into the pathophysiology of MI. Importantly, PTGDS changes at the earliest time compared to the three current biomarkers in both NSTEMI and STEMI patients (Fig. 4, *C* and *D*), suggesting that PTGDS may serve as an early diagnostic biomarker at the very early stages of MI when the current biomarkers have not yet risen. This is particularly important for the diagnosis of NSTEMI, as these patients lack significant ECG changes, making biomarker-based diagnosis critically important. In this regard, new and potentially earlier biomarkers may play an extremely important role with greater clinical significance. Based on the present study, PTGDS could be developed as a novel biomarker for early diagnosis in patients with NSTEMI. However, further studies are needed to validate these findings and explore the potential utility of PTGDS in combination with other biomarkers.

The present study demonstrates that plasma PTGDS is a promising candidate for the early diagnosis of STEMI and NSTEMI. Using DIA proteomics and validation in an independent cohort, we identified PTGDS as a biomarker that increases significantly earlier than the current biomarkers. Its high sensitivity and specificity, particularly in patients with NSTEMI, highlight its potential clinical usefulness.

### Limitations

While the findings are promising, several limitations should be acknowledged: The study was conducted at a single center with a total of 386 patients. The results need to be validated in a large, multicenter cohort to assess generalizability and clinical applicability. The complexity of the comparison of the sensitivity and specificity of PTGDS to the current biomarkers indicates the necessity for further optimization and validation to enhance its diagnostic accuracy. While this study demonstrates the utility of DIA for biomarker discovery, it should be noted that the analysis was performed on a Q Exactive mass spectrometer, which has lower speed and resolution. This technical limitation inherently restricts the depth of proteome coverage achievable in plasma samples.

## Conclusions

The present study using DIA proteomics methods and further validation in a new cohort of patients discovered that in both STEMI and NSTEMI, plasma PTGDS increases at an earlier stage of onset time than the current biomarkers (hs-TnI, CK-MB, and Myo) with comparable sensitivity and specificity to those biomarkers. These findings strongly suggest that PTGDS has high potential to be developed as an early diagnostic biomarker for MI, particularly in NSTEMI patients. The discovery of novel biomarkers like PTGDS holds great clinical significance, especially for identifying high-risk NSTEMI patients at an early stage. However, further studies are needed to validate these findings and explore the potential utility of PTGDS in combination with existing biomarkers to improve diagnostic accuracy and patient outcomes.

## Data Availability

The mass spectrometry proteomics data have been deposited to the ProteomeXchange Consortium via the PRIDE partner repository with the dataset identifier PXD056463 and can be access with username: reviewer_pxd056463@ebi.ac.uk, password: lzYGHBFFbP0Z.

## Supplemental data

This article contains supplemental data (34, 35).

## Conflict of interest

The authors declare that they have no conflicts of interest with the contents of this article.

## Figures and Tables

**Supplementary Figure 1:**
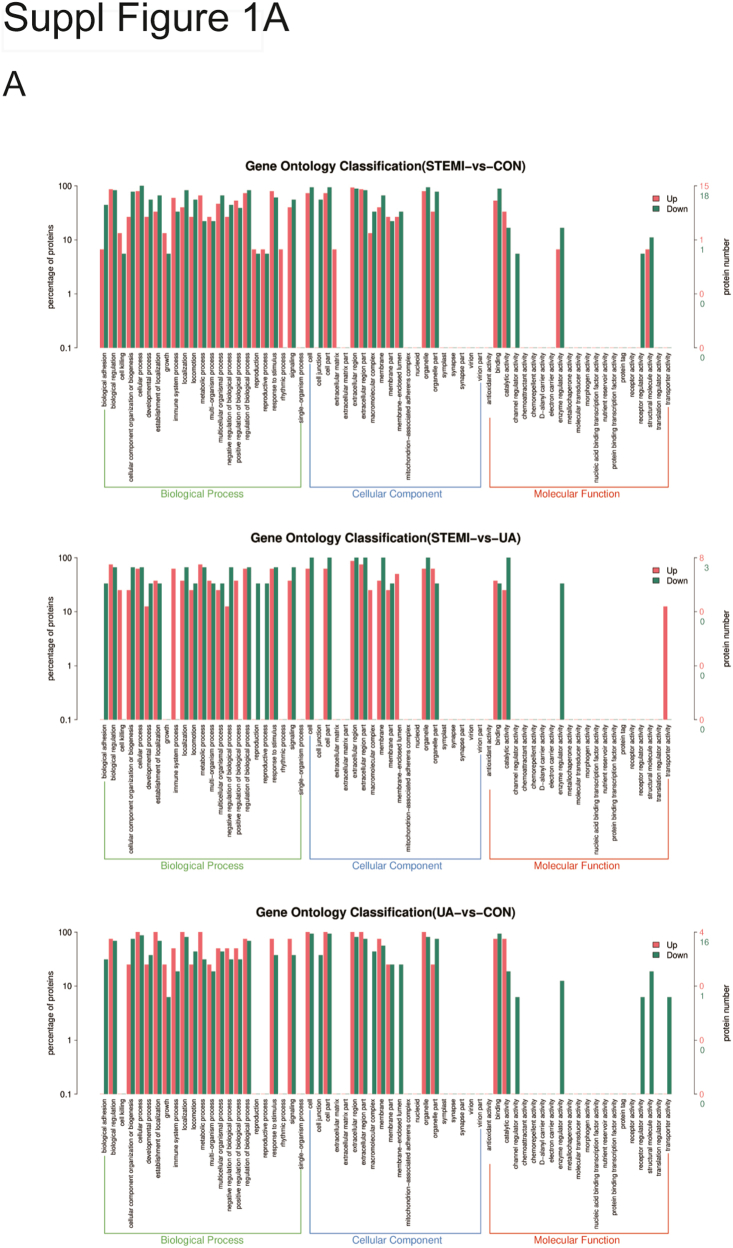
The Gene ontology (GO) level2 analysis of up- and down-regulated differentially abundant proteins (DAPs) and Kyoto Encyclopedia of Genes and Genomes (KEGG) pathway analysis of total proteins and DAPs.

**Supplementary Figure 1 continued:**
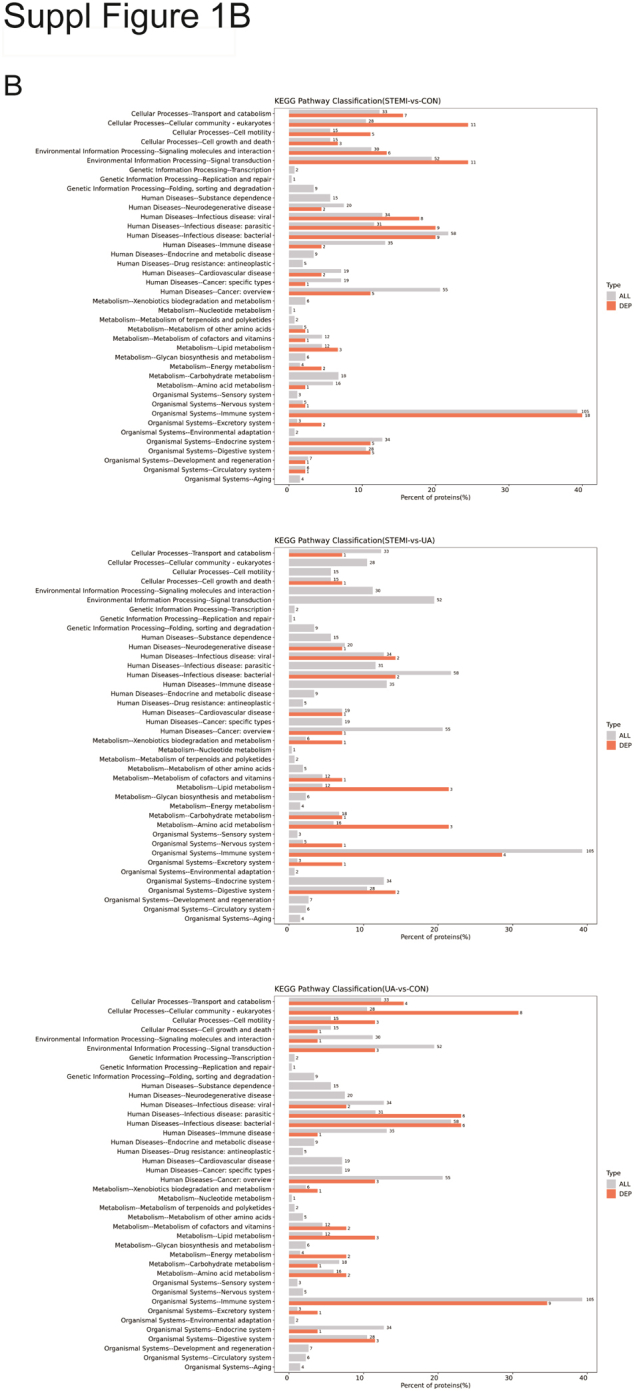


**Supplementary Figure 2:**
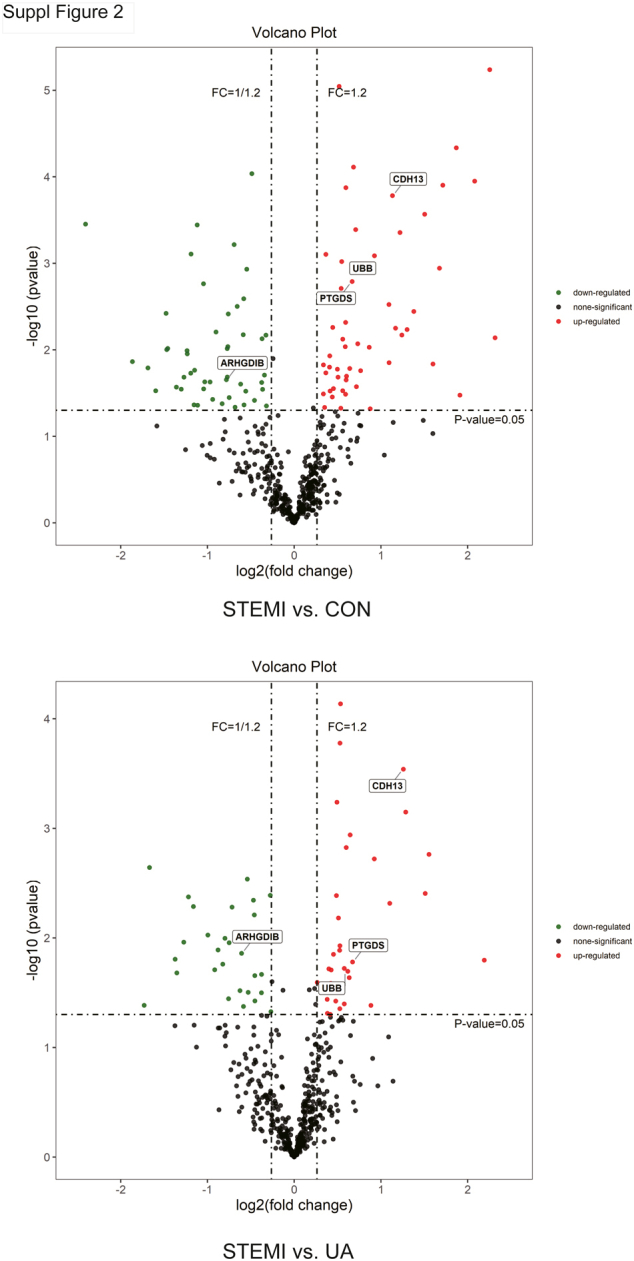
The location of the differentially abundant proteins (DAPs) in the volcano plot in comparison of STEMI *versus* CON or UA.

**Supplementary Figure 2 continued:**
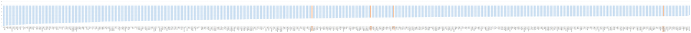


**Supplementary Figure 3:**
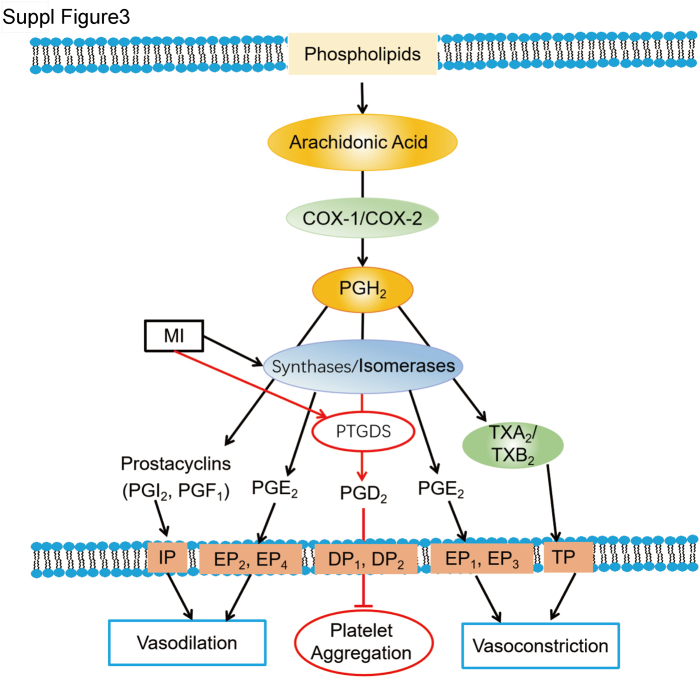
Schema of the possible metabolism pathway related to the increased PTGDS in MI.
